# Advancements in targeted therapies for acute myeloid leukemia

**DOI:** 10.1016/j.coph.2025.102604

**Published:** 2025-12-30

**Authors:** Matthew P. Connor, Adam Barsouk, Omar Elghawy, Catherine Lai

**Affiliations:** **Addresses**, University of Pennsylvania, Department of Medicine, Division of Hematology and Oncology, 3400 Civic Center Blvd, 12 South Pavilion, Philadelphia, PA 19104, USA

## Abstract

For decades, the only therapeutic option for acute myeloid leukemia (AML) had been intensive combination chemotherapy. In recent years, understanding of the molecular mechanisms underlying myeloid oncogenesis has grown immensely and has led to the development of multiple effective small molecule inhibitors of aberrant cellular signaling. This review highlights the major AML mutational pathways currently being targeted with precision therapies: the receptor tyrosine kinase FLT3, the citric acid cycle enzymes IDH1 and IDH2, and the transcription-regulating *KMT2A* and *NPM1* genes. We review the major clinical trials evaluating the safety and efficacy of agents targeting these pathways, as well as ongoing and upcoming studies of novel and combination therapies for these molecular subsets of AML.

## Introduction

Acute myeloid leukemia (AML) is an aggressive hematologic malignancy of hematopoietic precursors. For 40 years, intravenous cytarabine and daunorubicin (7 + 3) have remained the standard of care (SOC) for adults fit for intensive chemotherapy. Because induction therapy is highly toxic and alternatives for unfit, relapsed, or refractory patients are limited, novel AML treatment strategies are needed. Over the last two decades, understanding of hematopoiesis and tumor sequencing have transformed AML diagnosis and prognostication by identifying genetic drivers of myeloid oncogenesis [1,2]. Advanced understanding of molecular mechanisms has driven translational research targeting aberrant cellular pathways.

**Figure 1.**
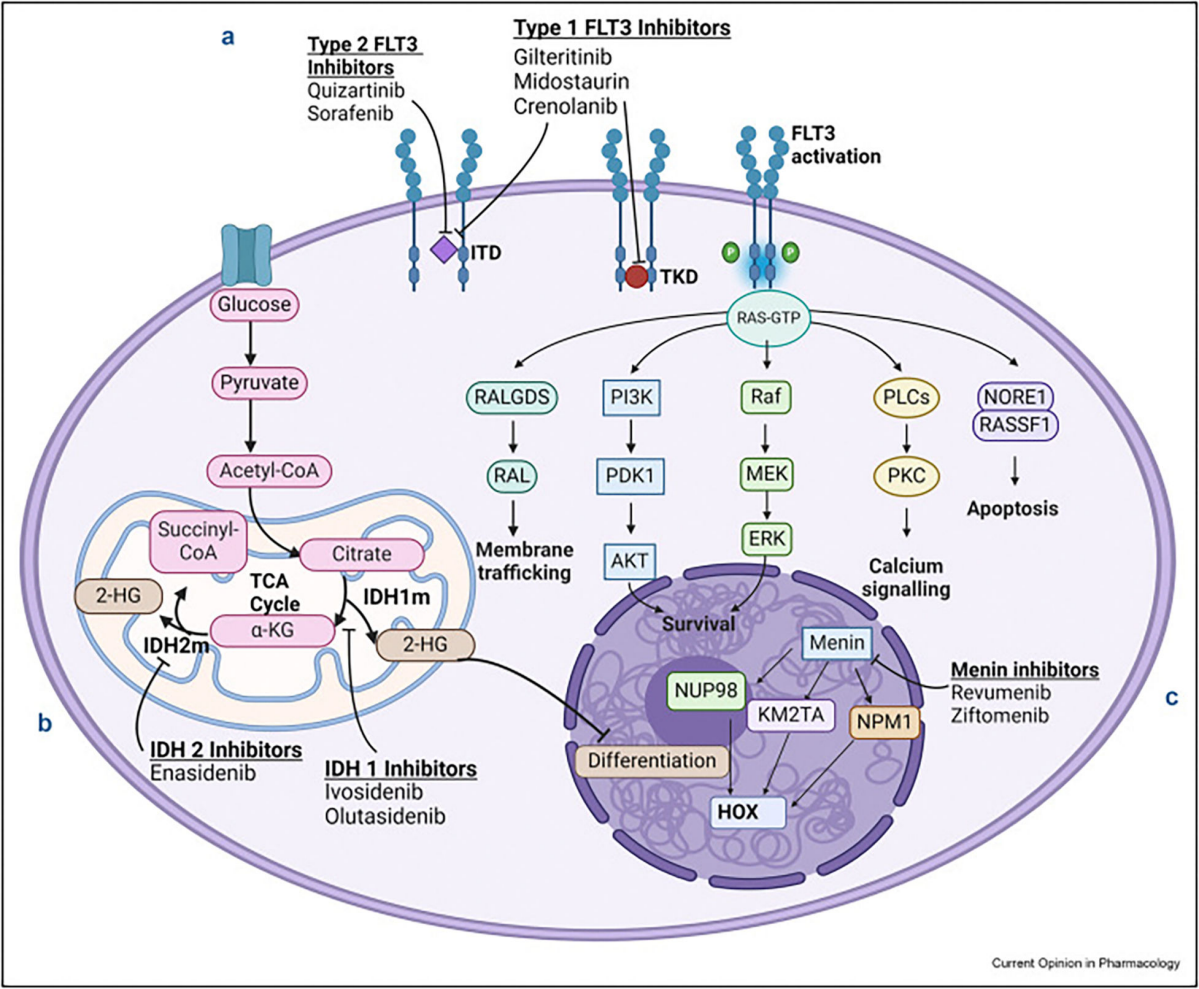
A hematopoietic progenitor cell with diagrammatic representations of 3 important signaling pathways mutated in molecular subtypes of AML. (**a**) The FLT3 receptor tyrosine kinase mediates signal transduction for multiple cell survival and proliferation mechanisms, including RAS/Raf/MAPK and PI3K/AKT pathways. Internal tandem duplication (ITD) in the juxtamembrane domain (most common) and mutations in the tyrosine kinase domain (TKD) lead to constitutive signaling and uncontrolled leukemic cell proliferation and survival – oncogenic effects that are blocked by FLT3 inhibitors. (**b**) IDH1 and IDH2 are citric acid cycle enzymes catalyzing the conversion of citrate to α-ketoglutarate (α-KG). Leukemogenic IDH1/2 mutations result in a reverse reaction, converting α-KG to the oncoprotein 2-hydroxyglutarate (2-HG) which interacts with epigenetic regulatory mechanisms to promote DNA and histone hypermethylation and inhibition of cellular differentiation. IDH1/2 inhibitors preferentially bind mutant IDH and reduce 2-HG production, promoting downstream differentiation. (**c**) *KMT2A* rearrangements and *NPM1* mutations in AML similarly increase *HOX* cluster and *MEIS1* gene expression, preventing differentiation and promoting survival. Menin is a key protein of *KMT2A* and *NPM1* gene product enzymatic complexes, and menin inhibitors reduce the activity of these aberrantly expressed mediators, successfully mitigating neoplastic self-renewal.

Novel therapies, including antibody drug conjugates (ADC) and small molecule inhibitors, are now integrated into AML therapy. Notable advancements include gemtuzumab ozogamicin (GO) for CD33-positive AML [3,4] and the BH3-mimetic venetoclax (VEN) with hypomethylating agents (HMA) for patients unfit for intensive induction [5]. Other precision therapeutics are now successfully targeting specific genomic subsets of AML. This review summarizes three of the major oncogenic mechanisms currently identified for intervention (Figure 1) and highlights seminal and ongoing clinical trials of targeted agents (Table 1).

## Current therapeutic targets

### FLT3

FMS-like tyrosine kinase 3 (*FLT3*) is a receptor tyrosine kinase that promotes hematopoietic cell survival and proliferation through multiple signaling pathways [6—8]. Mutations leading to constitutive signaling and blast proliferation are common in newly diagnosed (ND) AML and include internal tandem duplications (*FLT3-ITD,* ~30%) and tyrosine kinase domain mutations (*FLT3-TKD,* ~10%) [9,10]. *FLT3-ITD* AML historically confers inferior survival compared to wild-type *FLT3* AML [11,12], though its prognostic impact has evolved over time. Current European LeukemiaNet guidelines (2022) for genetic risk classify *FLT3-ITD* AML as intermediate risk absent additional adverse features [13]. This prognostic shift is attributable to introduction of FLT3 inhibitors (FLT3i) into frontline and salvage therapies with 3 agents currently approved by the U.S. Food and Drug Administration (FDA).

Midostaurin, a first-in-class multi-tyrosine kinase inhibitor (TKI), decreases FLT3 mutant autophosphorylation and inhibits both *ITD* and *TKD* mutations (a type 1 inhibitor) [14]. The phase 3 RATIFY trial randomized *FLT3* (*ITD* or *TKD*) patients <60 to midostaurin or placebo combined with 7 + 3 induction and high dose cytarabine (HiDAC) consolidation [15]. Though complete remission (CR) rates were similar (59% vs. 54%) with comparable toxicity, event-free survival (EFS) (8.2 vs. 3.0 months, p = 0.002) and median OS (mOS) (74.7 vs. 25.6 months, p = 0.009) were superior with midostaurin, leading to FDA approval during induction and consolidation. *FLT3-TKD* patients — who have better prognoses and comprised a large proportion of RATIFY subjects — derived greater EFS benefit from midostaurin compared to *FLT3-ITD* patients without differences in OS. RATIFY continued single agent midostaurin/placebo maintenance in responders, though was not designed to evaluate maintenance efficacy. Phase 2 studies [16—18] have shown potential, but not definitive benefit of maintenance midostaurin, which remains unlabeled for this indication. Long-term RATIFY follow-up has confirmed durable EFS and 10-year OS of 43.7% vs 38.6% with placebo (p = 0.0485) [19].

Quizartinib is a second-generation TKI with specificity for *FLT3-ITD* (a type 2 inhibitor). Avid receptor binding and slow dissociation leads to high signal inhibition, even in midostaurin-resistant cell lines [20]. The phase 3 QuANTUM-R trial demonstrated longer OS in R/R *FLT3-ITD* with quizartinib versus salvage chemotherapy (6.2 vs. 4.7 months, p = 0.02) [21], and the subsequent phase 3 QuANTUM-First trial established quizartinib’s upfront efficacy [22]. ND *FLT3-ITD* AML patients aged 18—75 (40% > 60) received quizartinib or placebo with induction 7 + 3, HiDAC consolidation, and up-to-3-year maintenance, irrespective of allogeneic hematopoietic cell transplant (HCT). Rates of CR/CR with incomplete hematologic recovery (CRi) (71.6% vs. 64.9%) and mOS (31.9 vs. 15.1 months) favored quizartinib over placebo. Neutropenia and QTc prolongation were more frequent in quizartinib-treated subjects. Quizartinib is FDA-approved for induction, consolidation, and maintenance. QuANTUM-First post-hoc analysis has shown OS benefit in those not undergoing HCT in first remission, and trend toward better OS in those who do [23]. As a type 2 inhibitor, quizartinib has limited efficacy against *FLT3-TKD* mutations; however, its high potency and utility as maintenance support its first-line use in *FLT3-ITD* patients without risk for QTc prolongation.

Gilteritinib, another second-generation, type 1 FLT3i, was the first targeted agent approved for R/R *FLT3* AML. In the phase 3 ADMIRAL study, gilteritinib improved CR/CR with partial (CRh) hematologic recovery (34.0% vs. 15.3%) and mOS (9.3 vs. 5.6 months) versus salvage chemotherapy [24,25]. Remissions are rarely durable. Thus, combination therapy is being actively investigated including with VEN (overall response rate (ORR) of 75%, mOS 10 months, 51% required dose interruptions for myelosuppression) [26] and with liposomal daunorubicin/cytarabine (CPX-351) (NCT05024552). Gilteritinib has shown efficacy as post-HCT maintenance therapy. The BMT CTN 1506/MORPHO phase 3 randomized *FLT3-ITD* patients post-HCT to 24 months of gilteritinib or placebo [27]. Two-year relapse-free survival (RFS) was 77.2% vs. 69.9% with placebo (p = 0.0518) with benefit driven by subjects with measurable residual disease (MRD) before or after HCT (hazard ratio 0.515, p = 0.0065). Phase 1 data suggest gilteritinib’s efficacy in ND *FLT3* AML with 35.8 month mOS combined with induction, consolidation, and as maintenance [28]. Ongoing phase 3 studies compare gilteritinib to midostaurin (NCT03836209, NCT04027309). We favor gilteritinib as a single-agent or with VEN in R/R *FLT3* AML or as post-HCT maintenance in patients not utilizing quizartinib.

No FLT3i is approved in patients with ND *FLT3* AML ineligible for intensive induction. Azacitidine (AZA) and VEN remains SOC based on the pivotal VIALE-A trial and pooled analysis demonstrated similar efficacy in wild-type and mutated *FLT3* [5,29]. A phase 1/2 trial of gilteritinib/AZA/VEN showed impressive CR rates with 18-month OS of 72%, however, myelotoxicity necessitated mitigative dosing strategies [30]. Trials are investigating this regimen (NCT04140487, NCT05520567) and triplet quizartinib/VEN/decitabine (NCT03661307) [31].

The type 2 TKI sorafenib can be considered for *FLT3-ITD* AML, per the National Comprehensive Cancer Network (NCCN) [32], for post-HCT maintenance [33,34] though has been largely replaced by more selective TKIs. The highly-selective type 1 FLT3i crenolanib is effective when added to induction for ND AML (86% ORR, 81% of CRs in molecular remission) [35]. mOS was not reached at 45 months follow-up, and a phase 3 study comparing crenolanib to midostaurin is enrolling (NCT03258931).

#### IDH1 and IDH2

Isocitrate dehydrogenase 1 (*IDH1*) and 2 (*IDH2*) are enzymes of the Krebs cycle. They catalyze α-ketoglutarate (α-KG) production and reduction of NADP+ to NADPH, playing roles in glucose metabolism and oxidative phosphorylation [36—38]. Mutant *IDH1* and *IDH2*, seen in 6—14% and 8—19% of AML, respectively [39—42], convert α-KG to the oncometabolite 2-hydroxyglutarate (2-HG). 2-HG inhibits catalytic activity and downstream demethylation, impairing blast differentiation [43]. The prognostic impact of *IDH* mutations is not fully understood; however, *IDH1*-mutant AML may be associated with poorer prognosis while *IDH2* may confer better outcomes [39,41,44,45].

*IDH* inhibitors bind to mutated catalytic sites, mostly sparing wild-type *IDH,* decreasing 2-HG production and promoting hematopoietic differentiation. *IDH* inhibitor therapy is associated with differentiation syndrome (DS) — characterized by fever, edema, pulmonary infiltrates, and end-organ damage — in up to 20% of patients [46]. DS necessitates close monitoring during therapy initiation and is treatable with glucocorticoids.

Ivosidenib is an *IDH1* inhibitor targeting mutations at the R132 locus [47]. It is approved monotherapy for R/R patients with a 30.4% CR/CRi rate and median duration of response (mDOR) of 6.5 months [48]. In ND *IDH1* AML ineligible for induction (median age 76.5 years) ivosidenib monotherapy has shown a 42.4% CR/CRh rate with mDOR not reached and mOS of 12.6 months [49]. Phase 1 data and the subsequent phase 3 AGILE trial studied combination ivosidenib/AZA in ND *IDH1* AML unfit for induction [50,51]. Ivosidenib/AZA had higher rates of CR/CRi (53% vs. 18%) with longer 12-month EFS (37% vs. 12%, p = 0.002), and mOS (24 vs. 7.9 months, p = 0.001) versus placebo/AZA. Rates of DS and grade ≥3 hematologic toxicities were relatively low. We consider ivosidenib/AZA as similarly effective frontline therapy to AZA/VEN [5], particularly in patients who are at high risk for hematologic toxicity. Ivosidenib can be considered for maintenance in *IDH1* AML for up to 1 year following HCT. In a phase 1 study, cumulative incidence of relapse (CIR, 19%) and non-relapse mortality (NRM, 0%) were low [52]. Ivosidenib is SOC therapy for *IDH1* AML patients with R/R disease and in those unfit for intensive induction with AZA or as monotherapy in frail patients. Studies of combinations with VEN ± HMA (NCT03471260, NCT04774393) and CPX-351 (NCT04493164) are underway.

Olutasidenib monotherapy is also approved for R/R *IDH1* AML based on phase 1/2 data showing a CR/CRi rate of 35% with mDOR of 25.3 months and mOS of 11.6 months [53—55]. Olutasidenib’s and ivosidenib’s toxicities differ slightly. QTc prolongation is more prominent with ivosidenib, hepatotoxicity with olutasidenib, and DS rates are similar [48,49,53,55]. Olutasidenib may effectively overcome ivosidenib resistance mutations [56]; however, no head-to-head data compares these agents. Ongoing trials are investigating olutasidenib for post-HCT maintenance (NCT06543381) and in combination for R/R or induction-ineligible patients (NCT06445959).

Enasidenib is a potent *IDH2* inhibitor targeting R140 and R172 variants now approved for R/R patients. Phase 1/2 data showed a 27.9% CR/CRi/CRp rate with mDOR and mOS of 5.6 and 8.8 months as monotherapy [57,58]. DS occurred at 9.6%. In a phase 1/2 trial in ND *IDH2* patients ≥60, enasidenib monotherapy achieved a 48% CR/CRi rate with 17.1-month mOS. AZA was added if CR/CRi was not achieved after 5 cycles, and a further 41% of patients achieved CR/CRi [59,60]. A subsequent study of induction-ineligible patients demonstrated ORR of 74% vs. 36% (p = 0.0003) with enasidenib/AZA versus AZA alone, but mOS was similar between groups (22.0 vs. 22.3 months, p = 0.97) [61]. Thus, enasidenib is only approved for R/R *IDH2* AML. A phase 1 trial of post-HCT maintenance treated patients for up to 1 year and established enasidenib tolerability with 16% CIR and NRM [62]. Two additional studies are investigating enasidenib maintenance (NCT04522895, NCT03728335). We often favor AZA/VEN as highly effective frontline therapy for *IDH2* AML, reserving enasidenib for R/R disease. However, given enasidenib’s potency, we do consider escalation to triplet therapy in highly selected patients who do not achieve early remissions with AZA/VEN and who plan to proceed to HCT.

Ivosidenib and enasidenib are also being evaluated in combination with induction therapy. Addition of ivosidenib or enasidenib to 7 + 3 and HiDAC consolidation with continuation maintenance showed tolerability and CR/CRi/CRp rates of 77% and 74%, in a phase 1 study. mOS was not reached with median follow-up of 21.2 (ivosidenib) and 23.7 months (enasidenib) [63,64]. The randomized phase 3 HOVON140AML trial comparing ivosidenib/enasidenib versus placebo during induction/consolidation/maintenance is enrolling in Europe and Australia (NCT03839771). AZA/VEN is also highly active in ND *IDH1* and *IDH2* AML with a composite CR rate of 79% and mOS of 24.5 months in pooled analysis of patients from VIALE-A and a phase Ib study of VEN + AZA or decitabine [65,66]. The ongoing phase 2 I-DATA trial randomizes ND *IDH1/IDH2* patients to either AZA/VEN or AZA/IDHi (ivosidenib or enasidenib). Subjects then receive the unassigned treatment arm at time of relapse/refractory disease, thus informing sequencing approaches of AZA/VEN versus *IDH*-targeted frontline therapy [67].

### KMT2A and NPM1

Epigenetic transcriptional regulation is important in hematopoiesis. Mutations in such pathways drive myeloid and lymphoid acute leukemias [68—70]. The histone-lysine *N*-methyltransferase 2A gene located on chromosome 11q23 is rearranged (*KMT2A-r)* in up to 10% of AML — an aberration often associated with prior topoisomerase II inhibitor exposure and associated with poor prognosis [44,71]. Anomalous downstream transcriptional checkpoint abnormalities upregulate homeobox (*HOX*) cluster genes and the DNA-binding cofactor *MEIS1*, causing arrest of stem cell differentiation. The KMT2A enzymatic complex includes the tumor suppressor protein menin, which binds to both wild-type and mutant KMT2A [72]. Menin inhibition nullifies aberrant oncogene overexpression in *KMT2A-r* cells [72].

Nucleophosmin (NPM) is a nuclear phosphoprotein important in ribosomal biogenesis, chromatin remodeling, and DNA repair [73]. Mutations in the *NPM1* gene (*NPM1-m*) result in irregular cytoplasmic localization of mutant NPM and upregulation of *HOX* genes similar to that seen in *KMT2A-r*. *NPM1-m* occur in ~30% of ND AML [74—76]. While incompletely described mechanistically, *NPM1-m* AML models show abatement of self-renewal signals with menin inhibition [77]. Thus, *KMT2A-r* and *NPM1-m* are now major targets for small molecule inhibitors.

Revumenib is a selective menin inhibitor studied in *KMT2A-r* and *NPM1-m* AML. In the AUGMENT-101 (NCT04065399) phase 1 trial, patients with R/R *KMT2A-r* or *NPM1-m* leukemias treated with revumenib had a CR/CRh/CRp rate of 38% with mDOR and mOS of 9.1 and 7.0 months, respectively [78]. Common toxicities were QTc prolongation (52.9%), nausea (26.5%), and DS (16.2%). Phase 2 of AUGMENT-101 found a 43.9% CR/CRh/CRi/CRp rate with 8.0 month mOS in *KMT2A-r* patients [79], and a 23.4% CR/CRh rate with 4.0 month mOS in *NPM1-m* patients [80]. QTc prolongation (25.5% in *KMT2A-r*, 40.5% in *NPM1-m*) and DS (27.7% in *KMT2A-r*, 17.9% in *NPM1-m*) remained prominent at the recommended phase 2 dose (RP2D), and post-HCT treatment continuation was permitted. Revumenib is now FDA approved for both R/R *KMT2A-*r and *NPM1-m* AML. A study of revumenib/AZA/VEN for ND *KMT2A-r* or *NPM1-m* AML found remarkably high rates of CR/CRh/CRi (100%) with 92% MRD-negative remissions and similar toxicity rates to AUGMENT-101 in 13 treated patients [81]. Other studies investigating revumenib in any *HOX*-upregulated leukemia (NCT06229912), in combination with other salvage (NCT05360160, NCT05326516, NCT06177067, NCT06222580) or frontline therapies (NCT06313437, NCT05886049, NCT06226571), and as maintenance after HCT (NCT06575296) are ongoing.

Ziftomenib is another menin inhibitor recently approved for R/R *NPM1-m* AML [82—84]. In the KOMET-001 trial, patients with R/R *KMT2A-r* or *NPM1-m* AML received ziftomenib monotherapy. Enrollment of *KMT2A-r* patients was halted due to high rates of DS. *NPM1-m* patients treated at the RP2D had a CR/CRh rate of 22% with mOS of 6.6 months [84]. Separate ongoing phase 1 studies combine ziftomenib with other salvage therapies (KOMET-008, NCT05735184) or with frontline chemotherapy in ND *KMT2A-r*/*NPM1-m* AML (KOMET-007, NCT05735184) with results eagerly awaited. Other menin inhibitors are under phase 1 investigation for R/R AML (NCT04811560, NCT05153330, NCT04988555, NCT04752163), highlighting the promise of this therapeutic target.

## Conclusion

Cytotoxic chemotherapy is effective in AML; however, therapy resistance, relapse, and toxicity are common obstacles. Genomic profiling has improved the understanding of myeloid oncogenesis, leading to an explosion of novel agents in a disease that has seen scarce therapeutic advancements for decades. *FLT3* inhibitors mitigate aberrant growth signaling leading to longer event-free and overall survival. *IDH* inhibitors promote leukemic blast differentiation and are effective as single agents for older adults or in combination therapy. New generations of *FLT3* and *IDH* inhibitors may reduce treatment-related toxicity and overcome resistance mechanisms. Menin inhibition is the next breakthrough in AML with multiple therapeutics imminently changing SOC for *KMT2A-r* and *NPM1-m* patients. Ongoing studies of future targets — such as treatment-resistant *TP53* mutations — are eagerly awaited. Other precision therapeutics such as monoclonal antibodies and adoptive cell therapies are still being developed. Targeted immunotherapies face multiple challenges in AML including antigen heterogeneity and myeloablative toxicities. Nevertheless, dozens of early phase studies targeting myeloid antigens such as CD33, CD70, CD123, and CD371 are currently underway for relapsed/refractory patients. Insights into leukemia genetics have redefined AML, and novel, personalized therapeutic strategies will evolve and improve outcomes for patients.

## Figures and Tables

**Table 1:** Summary of reported clinical trial data for select targeted AML therapies.

Target	Agent	Trial	Phase	Patients	Intervention	cCR^a^ (%)	OS (timepoint)	FDA Approval	Notes

*FLT3*	Mido	RATIFY [15]	3	ND, Ind eligible (n = 717)	Mido vs PLA with Ind/Con/Maint	58.9 vs 53.5	51.4% vs 44.3%^b^ (4-year)	Yes; With Ind/Con	Up to age 59; No Post-HCT maint
		AMLSG 16-10 [17, 18]	2	ND, Ind eligible (n = 440)	Mido with Ind/ Con/Maint	74.5	50.9% (2-year)	Yes; With Ind/Con	ITD only; up to 70 y post-HCT maint allowed
		RADIUS [16]	2	CR post-HCT (n = 60)	Mido vs SOC maint	NA	85% vs 76% (2-year)	No	ITD only; survival diff not significant
	Qui	QuANTUM-R [21]	3	R/R (n = 367)	Quiz vs salvage chemo	48.2 vs 27.0	27% vs 20%^b^ (1-year)	No	ITD only
		QuANTUM-first [22]	3	ND, Ind eligible (n = 539)	Quiz vs PLA with Ind/Cond/Maint	71.6 vs 64.9	31.9 vs 15.1 mo^b^ (median)	Yes; With Ind/Con/Maint	ITD only; up to age 75; post-HCT maint allowed
	Gilt	ADMIRAL [24,25]	3	R/R (n = 247)	Gilt vs salvage chemo	54.3 vs 21.8	37.1% vs 16.7%^b^ (1-year)	Yes; Alone for R/R	ITD or TKD
		M16-802 [26]	1 b	R/R (n = 61)	Gilt + ven	75	10.0 mo (median)	No	ITD or TKD
		MD ACC [30]	1/2	ND, unfit for Ind (n = 30) or R/R (n = 22)	Gilt + aza/Ven	96 (ND)27 (R/R)	83% (ND) 26% (R/R) (1-year)	No	ITD or TKD
		2215-CL-0103 [28]	1 b	ND, Ind eligible (n = 36)	Gilt with Ind/Con/Maint	89	46.1 (median months)	No	72% underwent HCT; Post-HCT maint allowed
		BMT CTN 1506/MORPHO [27]	3	CR post-HCT (n = 356)	Gilt vs PLA maint	NA	80.7% vs 77.5% (2-year)	No	ITD only; RFS 77.2% vs 69.9% (p = 0.0518) RFS benefit if MRD + before/after HCT (HR 0.515^b^)
	Cren	ARO-006 [35]	2	ND, Ind eligible (n = 44)	Cren with Ind/ Con/Maint	86	58.0% (3-year)	No	ITD or TKD; 81% molecular CR post-HCT maint allowed
	Sora	Sorafenib-Flt3 AML-2015 [34]	3	CR post-HCT (n = 202)	Sora vs No maint	NA	82.1% vs 68.0% (2-year)	No	ITD only; 12-month CIR 7.0% vs 24.5%^b^
		SORMAIN [33]	2	CR post-HCT (n = 83)	Sora vs PLA maint	NA	90.5% vs 66.2%^b^ (2-year)	No	ITD only; 24-month RFS 85.0% vs 53.3%^b^
*IDH1*	Ivo	AG120-C-001 [48]	1	R/R (n = 258)	Ivo	30.4	8.8 mo (median)	Yes; Alone for R/ R	mDOR 8.2 months
		AG120-C-001 [49]	1	ND, unfit for Ind (n = 34)	Ivo	42.4	12.6 mo (median)	Yes; Alone for ND in ≥75 or unfit for Ind	Median age 76.5
		AGILE [50]	3	ND, unfit for Ind (n = 146)	Ivo vs PLA + Aza	53 vs 18^b^	24.0 vs 7.9 mo^b^ (median)	Yes; +Aza for ND in ≥75 or unfit for Ind	EFS benefit in those ≥75 with low-intermediate risk
		MGH [52]	1	CR post-HCT (n = 16)	Ivo maint	NA	88% (2-year)	No	1-year maint; 2-year GRFS 25%
	Oluta	2102-HEM-101 [53]	1/2	R/R (n = 153)	Oluta	35	11.6 mo (median)	Yes; Alone for R/R	mDOR 25.9 mo.
*IDH2*	Ena	AG-221-C-001 [57, 58]	1/2	R/R (n = 280)	Ena	27.9	8.8 mo (median)	Yes; Alone for R/R	mDOR 5.6 mo.
		AG221-AML-005 [61]	2	ND, unfit for Ind (n = 101)	Ena vs PLA + Aza	74 vs 36b	72% vs 70% (1-year)	No	Median age 75; No significant OS/EFS difference
		Beat AML [60]	2/1 b	ND, unfit for Ind (n = 60)	Ena ± aza	48 (Ena) 60 (+Aza)	17 mo (Ena) 13 mo (+Aza) (median)	No	Median age 75
		MGH [62]	1	CR post-HCT (n = 19)	Ena maint	NA	74% (2-year)	No	1-year maint; 2-year CIR 16%; 42% mod/severe cGVHD
*IDH1/IDH2*		AG120-221-C-001 [63]	1	ND, Ind eligible (n = 153)	Ivo or ena with Ind/Con/Maint	77 (Ivo) 74 (Ena)	78% (Ivo) 76% (Ena) (1-year)	No	Median age 63; 46.4% went to HCT
*KMT2A/NPM1*	Rev	AUGMENT-101 [78]	1	R/R (n = 67 total; 56 AML)	Rev	38	7 mo (median)	No; Priority review	Median age 42.5; mDOR 9.1 mo.
		AUGMENT-101 [79, 80]	2	R/R (n = 94 *KMT2A* n = 84 *NPM1*)	Rev	*KMT2A* 43.9 *NPM1* 23.4	8 mo 4 mo (median)	Yes; Alone for R/R *KMT2A* or *NPM1*	mDOR 6.4 mo *(KMT2A)*, 4.7 mo (*NPM1*)
		Beat AML [81]	1 b	ND, unfit for Ind (n = 13)	Rev + aza/Ven	100	90% (1-year)	No	92% flow MRD-negative
	Zift	KOMET-001 [84]	2	R/R (n = 92)	Zift	22	6.6 mo (median)	Yes; Alone for *NPM1*	*KMT2A* enrollment halted at phase 1 for high ≥ Gr3 DS (28% vs 4%)

ITD, internal tandem duplication; TKD, tyrosine kinase domain; WT, wild-type; Mido, midostaurin; Qui, quizartinib; Gilt, gilteritinib; Cren, crenolanib; Sora, sorafenib; Ivo, ivosidenib; Oluta, olutasidenib; Ena, enasidenib; Rev, revumenib; Zift, ziftomenib; Ven, venetoclax; Aza, azacitidine; PLA, placebo; Ind, induction; Con, consolidation; Maint, maintenance; HCT, allogeneic hematopoietic cell transplant; R/R, relapsed/refractory; ND, newly diagnosed; CR, complete remission; CRi, complete remission with incomplete count recovery; CRh, complete remission with partial hematologic recovery; CRp, complete remission with incomplete platelet recovery; MLFS, morphologic leukemia-free state; RFS, relapse-free survival; PFS, progression-free survival; EFS, event-free survival; OS, overall survival; CIR, cumulative incidence of relapse; FDA, Food & Drug Administration; NCCN, National Comprehensive Cancer Network; MRD, measurable residual disease; GVHD, graft-versus-host disease; GRFS, GVHD-free and relapse-free survival; mDOR, median duration of response; DS, differentiation syndrome.

a cCR, composite complete response includes all patients achieving complete remission with or without incomplete or partial hematologic recovery (i.e., CR/CRi/CRh/CRp).

b Denotes statistically significant difference in values as specified by trial.
